# Waning 2-Dose and 3-Dose Effectiveness of mRNA Vaccines Against COVID-19–Associated Emergency Department and Urgent Care Encounters and Hospitalizations Among Adults During Periods of Delta and Omicron Variant Predominance — VISION Network, 10 States, August 2021–January 2022

**DOI:** 10.15585/mmwr.mm7107e2

**Published:** 2022-02-18

**Authors:** Jill M. Ferdinands, Suchitra Rao, Brian E. Dixon, Patrick K. Mitchell, Malini B. DeSilva, Stephanie A. Irving, Ned Lewis, Karthik Natarajan, Edward Stenehjem, Shaun J. Grannis, Jungmi Han, Charlene McEvoy, Toan C. Ong, Allison L. Naleway, Sarah E. Reese, Peter J. Embi, Kristin Dascomb, Nicola P. Klein, Eric P. Griggs, Deepika Konatham, Anupam B. Kharbanda, Duck-Hye Yang, William F. Fadel, Nancy Grisel, Kristin Goddard, Palak Patel, I-Chia Liao, Rebecca Birch, Nimish R. Valvi, Sue Reynolds, Julie Arndorfer, Ousseny Zerbo, Monica Dickerson, Kempapura Murthy, Jeremiah Williams, Catherine H. Bozio, Lenee Blanton, Jennifer R. Verani, Stephanie J. Schrag, Alexandra F. Dalton, Mehiret H. Wondimu, Ruth Link-Gelles, Eduardo Azziz-Baumgartner, Michelle A. Barron, Manjusha Gaglani, Mark G. Thompson, Bruce Fireman

**Affiliations:** ^1^CDC COVID-19 Emergency Response Team; ^2^School of Medicine, University of Colorado Anschutz Medical Campus, Aurora, Colorado; ^3^Center for Biomedical Informatics, Regenstrief Institute, Indianapolis, Indiana; ^4^Fairbanks School of Public Health, Indiana University, Indianapolis, Indiana; ^5^Westat, Rockville, Maryland; ^6^HealthPartners Institute, Minneapolis, Minnesota; ^7^Center for Health Research, Kaiser Permanente Northwest, Portland, Oregon; ^8^Kaiser Permanente Vaccine Study Center, Kaiser Permanente Northern California Division of Research, Oakland, California; ^9^Department of Biomedical Informatics, Columbia University Irving Medical Center, New York, New York; ^10^New York Presbyterian Hospital, New York, New York; ^11^Division of Infectious Diseases and Clinical Epidemiology, Intermountain Healthcare, Salt Lake City, Utah; ^12^Indiana University School of Medicine, Indianapolis, Indiana; ^13^Vanderbilt University Medical Center, Nashville, Tennessee; ^14^Baylor Scott & White Health, Temple, Texas; ^15^Children's Minnesota, Minneapolis, Minnesota; ^16^Texas A&M University College of Medicine, Temple, Texas.

CDC recommends that all persons aged ≥12 years receive a booster dose of COVID-19 mRNA vaccine ≥5 months after completion of a primary mRNA vaccination series and that immunocompromised persons receive a third primary dose.* Waning of vaccine protection after 2 doses of mRNA vaccine has been observed during the period of the SARS-CoV-2 B.1.617.2 (Delta) variant predominance^†^ (*1*–*5*), but little is known about durability of protection after 3 doses during periods of Delta or SARS-CoV-2 B.1.1.529 (Omicron) variant predominance. A test-negative case-control study design using data from eight VISION Network sites^§^ examined vaccine effectiveness (VE) against COVID-19 emergency department/urgent care (ED/UC) visits and hospitalizations among U.S. adults aged ≥18 years at various time points after receipt of a second or third vaccine dose during two periods: Delta variant predominance and Omicron variant predominance (i.e., periods when each variant accounted for ≥50% of sequenced isolates).^¶^ Persons categorized as having received 3 doses included those who received a third dose in a primary series or a booster dose after a 2 dose primary series (including the reduced-dosage Moderna booster). The VISION Network analyzed 241,204 ED/UC encounters** and 93,408 hospitalizations across 10 states during August 26, 2021–January 22, 2022. VE after receipt of both 2 and 3 doses was lower during the Omicron-predominant than during the Delta-predominant period at all time points evaluated. During both periods, VE after receipt of a third dose was higher than that after a second dose; however, VE waned with increasing time since vaccination. During the Omicron period, VE against ED/UC visits was 87% during the first 2 months after a third dose and decreased to 66% among those vaccinated 4–5 months earlier; VE against hospitalizations was 91% during the first 2 months following a third dose and decreased to 78% ≥4 months after a third dose. For both Delta- and Omicron-predominant periods, VE was generally higher for protection against hospitalizations than against ED/UC visits. All eligible persons should remain up to date with recommended COVID-19 vaccinations to best protect against COVID-19–associated hospitalizations and ED/UC visits.

VISION Network methods have been previously published (*6*). Eligible medical encounters were defined as those among adults aged ≥18 years with a COVID-19–like illness diagnosis^††^ who had received molecular testing (primarily reverse transcription–polymerase chain reaction assay) for SARS-CoV-2, the virus that causes COVID-19, during the 14 days before through 72 hours after the medical encounter. The study period began on August 26, 2021, 14 days after the first U.S. recommendation for a third mRNA COVID-19 vaccine dose.^§§^ The date when the Omicron variant accounted for ≥50% of sequenced isolates was determined for each study site based on state and national surveillance data. Recipients of Ad.26.COV2.S (Janssen [Johnson & Johnson]) vaccine, 1 or >3 doses of an mRNA vaccine, and those for whom <14 days had elapsed since receipt of any dose were excluded.

VE was estimated using a test-negative design, comparing the odds of a positive SARS-CoV-2 test result between vaccinated and unvaccinated patients using logistic regression models conditioned on calendar week and geographic area and adjusting for age, local virus circulation, immunocompromised status, additional patient comorbidities, and other patient and facility characteristics.^¶¶^ Immunocompromised status was identified by previously published diagnosis codes.*** Vaccination status was categorized based on the number of vaccine doses received and number of days between receipt of the most recent vaccine dose and the index medical encounter date (referred to as time since vaccination).^†††^ Patients with no record of mRNA vaccination before the index date were considered unvaccinated. Persons categorized as having received 3 doses included those who received a third dose in a primary series or a booster dose after a 2 dose primary series (including the reduced-dosage Moderna booster).

A standardized mean or proportion difference ≥0.2 indicated a nonnegligible difference in distributions of vaccination or infection status. The most remote category of time since vaccination was either ≥4 months or ≥5 months, depending on data availability (no hospitalizations were observed ≥5 months after receipt of a third dose during either period). To test for a trend in waning, time since vaccination categories were specified as an ordinal variable (<2 months = 0; 2–3 months = 1; 4 months = 2; ≥5 months = 3), with statistically significant waning indicated by a p-value <0.05 for the resulting regression coefficient. SAS (version 9.4, SAS Institute) and R software (version 4.1.2, R Foundation) were used to prepare data and perform statistical analysis.

For illustration purposes, the earliest and latest VE estimates for the trend are described. The overall trend can be statistically significant even though the precision of each estimate might be low, with the 95% CIs of estimates including zero. Analyses were stratified by two periods: Delta variant predominance and Omicron variant predominance. This study was reviewed and approved by the institutional review boards at participating sites and under a reliance agreement with the Westat, Inc. Institutional Review Board.^§§§^

Among 241,204 eligible ED/UC encounters, 185,652 (77%) and 55,552 (23%) occurred during the Delta- and Omicron-predominant periods, respectively (Table 1). Among persons with COVID-19–like illness seeking care at ED/UC facilities, 46% were unvaccinated, 44% had received 2 doses of vaccine, and 10% had received 3 doses. The median interval since receipt of the most recent dose before the ED/UC encounter was 214 days (IQR = 164–259 days) among those who had received 2 doses and 49 days (IQR = 30–73) among those who had received 3 doses (CDC, unpublished data, 2022).

**TABLE 1:** Characteristics of emergency department and urgent care encounters among adults with COVID-19–like illness,* by mRNA COVID-19 vaccination status^†^ and SARS-CoV-2 test result — 10 states,^§^ August 2021–January 2022^¶^

Characteristic	Total no. (column %)	mRNA COVID-19 vaccination status no. (row %)			SMD^††^	SARS-CoV-2 test result no. (row %)		SMD^††^
		Unvaccinated	Vaccinated (2 doses)	Vaccinated (3 doses)**		Negative	Positive	
All ED/UC encounters	241,204 (100)	110,873 (46)	105,193 (44)	25,138 (10)	—	179,378 (74)	61,826 (26)	—
**Variant predominance period**								
B.1.617.2 (Delta)	**185,652 (77)**	86,074 (46)	85,371 (46)	14,207 (8)	0.27	148,106 (80)	37,546 (20)	0.50
B.1.1.529 (Omicron)	**55,552 (23)**	24,799 (45)	19,822 (36)	10,931 (20)		31,272 (56)	24,280 (44)	
**Site**								
Baylor Scott & White Health	**40,621 (17)**	23,827 (59)	14,438 (36)	2,356 (6)	0.70	28,701 (71)	11,920 (29)	0.40
Columbia University^§§^	**5,681 (2)**	3,039 (53)	2,388 (42)	254 (4)		4,025 (71)	1,656 (29)	
HealthPartners^§§^	**4,893 (2)**	1,352 (28)	3,270 (67)	271 (6)		4,109 (84)	784 (16)	
Intermountain Healthcare	**61,333 (25)**	25,072 (41)	29,407 (48)	6,854 (11)		50,637 (83)	10,696 (17)	
Kaiser Permanente Northern California	**45,753 (19)**	11,165 (24)	25,335 (55)	9,253 (20)		34,715 (76)	11,038 (24)	
Kaiser Permanente Northwest	**16,625 (7)**	5,895 (35)	8,620 (52)	2,110 (13)		13,561 (82)	3,064 (18)	
Regenstrief Institute	**41,694 (17)**	26,799 (64)	12,541 (30)	2,354 (6)		25,420 (61)	16,274 (39)	
University of Colorado	**24,604 (10**)	13,724 (56)	9,194 (37)	1,686 (7)		18,210 (74)	6,394 (26)	
**Age group, yrs**								
18–44	**110,203 (46)**	65,073 (59)	40,936 (37)	4,194 (4)	0.81	80,085 (73)	30,118 (27)	0.23
45–64	**64,583 (27)**	28,479 (44)	30,272 (47)	5,832 (9)		45,710 (71)	18,873 (29)	
65–74	**31,172 (13)**	9,390 (30)	15,289 (49)	6,493 (21)		24,304 (78)	6,868 (22)	
75–84	**23,242 (10)**	5,360 (23)	12,160 (52)	5,722 (25)		19,155 (82)	4,087 (18)	
≥85	**12,004 (5)**	2,571 (21)	6,536 (54)	2,897 (24)		10,124 (84)	1,880 (16)	
**Sex**								
Male^¶¶^	**97,859 (41)**	47,368 (48)	40,062 (41)	10,429 (11)	0.06	70,430 (72)	27,429 (28)	0.10
Female	**143,345 (59)**	63,505 (44)	65,131 (45)	14,709 (10)		108,948 (76)	34,397 (24)	
**Race/Ethnicity**								
White, non-Hispanic	**150,419 (62)**	65,355 (43)	67,433 (45)	17,631 (12)	0.30	116,134 (77)	34,285 (23)	0.22
Hispanic	**37,043 (15)**	18,238 (49)	16,054 (43)	2,751 (7)		26,148 (71)	10,895 (29)	
Black, non-Hispanic	**24,702 (10)**	14,633 (59)	8,653 (35)	1,416 (6)		16,534 (67)	8,168 (33)	
Other, non-Hispanic***	**17,683 (7)**	6,153 (35)	9,009 (51)	2,521 (14)		13,360 (76)	4,323 (24)	
Unknown	**11,357 (5)**	6,494 (57)	4,044 (36)	819 (7)		7,202 (63)	4,155 (37)	
**Chronic respiratory condition^†††^**								
Yes^¶¶^	**42,531 (18)**	17,884 (42)	19,359 (46)	5,288 (12)	0.09	35,264 (83)	7,267 (17)	0.22
No	**198,673 (82)**	92,989 (47)	85,834 (43)	19,850 (10)		144,114 (73)	54,559 (27)	
**Chronic nonrespiratory condition^§§§^**								
Yes^¶¶^	**62,192 (26)**	24,884 (40)	29,202 (47)	8,106 (13)	0.17	50,304 (81)	11,888 (19)	0.21
No	**179,012 (74)**	85,989 (48)	75,991 (42)	17,032 (10)		129,074 (72)	49,938 (28)	
**Immunocompromised status^¶¶¶^**								
Yes^¶¶^	**9,546 (4)**	3,348 (35)	4,462 (47)	1,736 (18)	0.12	8,222 (86)	1,324 (14)	0.14
No	**231,658 (96)**	107,525 (46)	100,731 (43)	23,402 (10)		171,156 (74)	60,502 (26)	
**Total vaccinated**	**130,331 (54)**	—	105,193 (81)	25,138 (19)		111,559 (86)	18,772 (14)	
**Vaccine product**								
Pfizer-BioNTech	**79,806 (61)**	—	63,912 (80)	15,894 (20)	—	67,179 (84)	12,627 (16)	0.15
Moderna	**48,990 (38)**	—	41,046 (84)	7,944 (16)		42,980 (88)	6,010 (12)	
Combination of mRNA products	**1,535 (1)**	—	235 (15)	1,300 (85)		1,400 (91)	135 (9)	
**No. of doses received (interval from receipt of most recent dose to ED/UC encounter)**								
2 (<2 mos)	**4,808 (4)**	—	4,808 (100)	—	—	4,507 (94)	301 (6)	0.38
2 (2–3 mos)	**10,644 (8)**	—	10,644 (100)	—		9,332 (88)	1,312 (12)	
2 (4 mos)	**10,175 (8)**	—	10,175 (100)	—		8,945 (88)	1,230 (12)	
2 (≥5 mos)	**79,566 (61)**	—	79,566 (100)	—		65,922 (83)	13,644 (17)	
3 (<2 mos)	**15,614 (12)**	—	—	15,614 (100)		14,694 (94)	920 (6)	
3 (2–3 mos)	**8,759 (7)**	—	—	8,759 (100)		7,639 (87)	1,120 (13)	
3 (4 mos)	**736 (1)**	—	—	736 (100)		509 (69)	227 (31)	
3 (≥5 mos)	**29 (0)**	—	—	29 (100)		11 (38)	18 (62)	

**Abbreviations**: ED = emergency department; ICD-9 = *International Classification of Diseases, Ninth Revision*; ICD-10 = *International Classification of Diseases, Tenth Revision* SMD = standardized mean or proportion difference; UC = urgent care.

* Medical events with a discharge code consistent with COVID-19–like illness were included. COVID-19–like illness diagnoses included acute respiratory illness (e.g., COVID-19, respiratory failure, or pneumonia) or related signs or symptoms (cough, fever, dyspnea, vomiting, or diarrhea) using ICD-9 and ICD-10 diagnosis codes. Clinician-ordered molecular assays (e.g., real-time reverse transcription–polymerase chain reaction) for SARS-CoV-2 occurring ≤14 days before to <72 hours after admission were included. Recipients of Janssen vaccine, 1 or >3 doses of an mRNA vaccine, and those for whom 1–13 days had elapsed since receipt of any dose were excluded.

^†^ Vaccination was defined as having received the listed number of doses of an mRNA-based COVID-19 vaccine ≥14 days before the medical event index date, which was the date of respiratory specimen collection associated with the most recent positive or negative SARS-CoV-2 test result before medical event or the admission date if testing only occurred after the admission.

^§^ California, Colorado, Indiana, Minnesota, New York, Oregon, Texas, Utah, Washington, and Wisconsin.

^¶^ Partners contributing data on medical events and estimated date of Omicron predominance were in California (December 21), Colorado (December 19), Indiana (December 26), Minnesota and Wisconsin (December 25), New York (December 18), Oregon (December 24), Texas (December 16), Utah (December 24), and Washington (December 24). The study period began in September 2021 for partners located in Texas.

** The ”Vaccinated (3 doses)” category includes persons who have received a third dose in their primary series or have received a booster dose following their 2-dose primary series; the third dose could have been either a 100-*μ*g or 50-*μ*g dose of Moderna vaccine or a 30-*μ*g dose of the Pfizer-BioNTech vaccine.

^††^ An absolute SMD ≥0.20 indicates a nonnegligible difference in variable distributions between medical events for vaccinated versus unvaccinated patients. When calculating SMDs for differences in characteristics across mRNA COVID-19 vaccination status, the SMD was calculated as the average of the absolute value of the SMD for unvaccinated versus vaccinated with 2 doses and the absolute value of the SMD for unvaccinated versus vaccinated with 3 doses. All SMDs are reported as the absolute SMD.

^§§^ ED data at Columbia University Irving Medical Center and HealthPartners exclude encounters that were transferred to an inpatient setting

^¶¶^ Referent group used for SMD calculations for dichotomous variables.

*** Other race includes Asian, Native Hawaiian or other Pacific islander, American Indian or Alaska Native, Other not listed, and multiple races.

^†††^ Chronic respiratory condition was defined using ICD-9 and ICD-10 as the presence of discharge codes for asthma, chronic obstructive pulmonary disease, or other lung disease.

^§§§^ Chronic nonrespiratory condition was defined using ICD-9 and ICD-10 as the presence of discharge codes for heart failure, ischemic heart disease, hypertension, other heart disease, stroke, other cerebrovascular disease, diabetes type I or II, other diabetes, metabolic disease, clinical obesity, clinically underweight, renal disease, liver disease, blood disorder, immunosuppression, organ transplant, cancer, dementia, neurologic disorder, musculoskeletal disorder, or Down syndrome.

^¶¶¶^ Immunocompromised status was defined using ICD-9 and ICD-10 as the presence of discharge codes for solid malignancy, hematologic malignancy, rheumatologic or inflammatory disorder, other intrinsic immune condition or immunodeficiency, or organ or stem cell transplant.

During the Delta-predominant period, VE against laboratory-confirmed COVID-19–associated ED/UC encounters was higher after receipt of a third dose than after a second dose; however, VE declined with increasing time since vaccination (Table 2). Among recipients of 3 doses, VE was 97% within 2 months of vaccination and declined to 89% among those vaccinated ≥4 months earlier (p<0.001 for test of trend in waning VE).

**TABLE 2:** mRNA COVID-19 vaccine effectiveness* against laboratory-confirmed COVID-19–associated^†^ emergency department and urgent care encounters and hospitalizations among adults aged ≥18 years, by number and timing of vaccine doses^§^ — VISION Network, 10 states,^¶^ August 2021–January 2022**

**Characteristic**	**Total**	**SARS-CoV-2 positive test result no. (%)**	VE fully adjusted % (95% CI)*	**Waning trend p value^††^**
**ED/UC encounters**				
**Overall**				
Unvaccinated (Ref)	**110,873**	43,054 (39)	—	—
**Any mRNA vaccine, 2 doses**	**105,193**	16,487 (16)	72 (72–73)	<0.001
<2 mos	**4,808**	301 (6)	88 (87–90)	
2–3 mos	**10,644**	1,312 (12)	80 (78–81)	
4 mos	**10,175**	1,230 (12)	79 (77–80)	
≥5 mos	**79,566**	13,644 (17)	69 (68–70)	
**Any mRNA vaccine, 3 doses**	**25,138**	2,285 (9)	89 (89–90)	<0.001
<2 mos	**15,614**	920 (6)	92 (91–93)	
2–3 mos	**8,759**	1,120 (13)	86 (85–87)	
4 mos	**736**	227 (31)	75 (70–79)	
≥5 mos	**29**	18 (62)	50 (-7–77)	
**Delta-predominant period**				
Unvaccinated (Ref)	**86,074**	29,063 (34)	—	—
**Any mRNA vaccine, 2 doses**	**85,371**	8,136 (10)	80 (79–81)	<0.001
<2 mos	**4,253**	144 (3)	92 (91–94)	
2–3 mos	**8,662**	527 (6)	88 (86–89)	
4 mos	**8,941**	721 (8)	85 (83–86)	
≥5 mos	**63,515**	6,744 (11)	77 (76–78)	
**Any mRNA vaccine, 3 doses**	**14,207**	347 (2)	96 (95–96)	<0.001
<2 mos	**10,621**	210 (2)	97 (96–97)	
2–3 mos	**3,542**	134 (4)	93 (92–94)	
≥4 mos	**44**	3 (7)	89 (64–97)	
**Omicron-predominant period**				
Unvaccinated (Ref)	**24,799**	13,991 (56)	—	—
**Any mRNA vaccine, 2 doses**	**19,822**	8,351 (42)	41 (38–43)	<0.001
<2 mos	**555**	157 (28)	69 (62–75)	
2–3 mos	**1,982**	785 (40)	50 (45–55)	
4 mos	**1,234**	509 (41)	48 (41–54)	
≥5 mos	**16,051**	6,900 (43)	37 (34–40)	
**Any mRNA vaccine, 3 doses**	**10,931**	1,938 (18)	83 (82–84)	<0.001
<2 mos	**4,993**	710 (14)	87 (85–88)	
2–3 mos	**5,217**	986 (19)	81 (79–82)	
4 mos	**692**	224 (32)	66 (59–71)	
≥5 mos	**29**	18 (62)	31 (−50–68)	
**Hospitalizations**				
**Overall**				
Unvaccinated (Ref)	**40,125**	16,335 (41)	—	—
**Any mRNA vaccine, 2 doses**	**42,326**	4,294 (10)	82 (81–83)	<0.001
<2 mos	**1,662**	71 (4)	93 (91–94)	
2–3 mos	**3,084**	223 (7)	88 (86–90)	
4 mos	**3,279**	234 (7)	89 (87–90)	
≥5 mos	**34,301**	3,766 (11)	80 (79–81)	
**Any mRNA vaccine, 3 doses**	**10,957**	471 (4)	93 (92–94)	<0.001
<2 mos	**7,332**	221 (3)	95 (94–95)	
2–3 mos	**3,413**	211 (6)	91 (89–92)	
≥4 mos	**212**	39 (18)	81 (72–87)	
**Delta-predominant period**				
Unvaccinated (Ref)	**36,214**	14,445 (40)	—	—
**Any mRNA vaccine, 2 doses**	**38,707**	3,315 (9)	85 (84–85)	<0.001
<2 mos	**1,574**	49 (3)	94 (92–96)	
2–3 mos	**2,790**	154 (6)	91 (89–92)	
4 mos	**3,129**	192 (6)	90 (89–92)	
≥5 mos	**31,214**	2,920 (9)	82 (82–83)	
**Any mRNA vaccine, 3 doses**	**8,124**	195 (2)	95 (95–96)	<0.001
<2 mos	**6,071**	118 (2)	96 (95–97)	
2–3 mos	**2,030**	74 (4)	93 (91–95)	
≥4 mos	**23**	3 (13)	76 (14–93)	
**Omicron-predominant period**				
Unvaccinated (Ref)	**3,911**	1,890 (48)	—	—
**Any mRNA vaccine, 2 doses**	**3,619**	979 (27)	55 (50–60)	0.01
<2 mos	**88**	22 (25)	71 (51–83)	
2–3 mos	**294**	69 (23)	65 (53–74)	
4 mos	**150**	42 (28)	58 (38–71)	
≥5 mos	**3,087**	846 (27)	54 (48–59)	
**Any mRNA vaccine, 3 doses**	**2,833**	276 (10)	88 (86–90)	<0.001
<2 mos	**1,261**	103 (8)	91 (88–93)	
2–3 mos	**1,383**	137 (10)	88 (85–90)	
≥4 mos	**189**	36 (19)	78 (67–85)	

**Abbreviations:** ED = emergency department; ICD-9 = *International Classification of Diseases, Ninth Revision*; ICD-10 *= International Classification of Diseases, Tenth Revision*; Ref = referent group; UC = urgent care; VE = vaccine effectiveness.

* VE was calculated as [1 − odds ratio] x 100%, estimated using a test-negative design, conditioned on calendar week and geographic area, and adjusted for age, local virus circulation (percentage of SARS-CoV-2–positive results from testing within the counties surrounding the facility on the date of the encounter), propensity to be vaccinated (calculated separately for each VE estimate), and other factors. Generalized boosted regression tree methods were used to estimate the propensity to be vaccinated based on sociodemographic characteristics, underlying medical conditions, and facility characteristics.

^†^ Medical events with a discharge code consistent with COVID-19–like illness were included. COVID-19–like illness diagnoses included acute respiratory illness (e.g., COVID-19, respiratory failure, or pneumonia) or related signs or symptoms (cough, fever, dyspnea, vomiting, or diarrhea) using ICD-9 and ICD-10 diagnosis codes. Clinician-ordered molecular assays (e.g., real-time reverse transcription–polymerase chain reaction) for SARS-CoV-2 occurring ≤14 days before to <72 hours after admission were included. Recipients of Janssen vaccine, 1 or >3 doses of an mRNA vaccine, and those for whom <14 days had elapsed since receipt of any dose were excluded.

^§^ Vaccination status was documented in electronic health records and immunization registries and was defined as having received the listed number of doses of an mRNA-based COVID-19 vaccine ≥14 days before the medical event index date. Index date was defined as the date of respiratory specimen collection associated with the most recent positive or negative SARS-CoV-2 test result before the medical event or the admission date if testing only occurred after the admission. Persons categorized as having received 3 vaccine doses include those who received a third dose in their primary series or received a booster dose after their 2 dose primary series; the third dose could have been either a 100-*μ*g or 50-*μ*g dose of Moderna vaccine or a 30-*μ*g dose of the Pfizer-BioNTech vaccine.

^¶^ California, Colorado, Indiana, Minnesota, New York, Oregon, Texas, Utah, Washington, and Wisconsin.

** Partners contributing data on medical events and estimated dates of Omicron predominance were in California (December 21), Colorado (December 19), Indiana (December 26), Minnesota and Wisconsin (December 25), New York (December 18), Oregon (December 24), Texas (December 16), Utah (December 24), and Washington (December 24). The study period began in September 2021 for partners located in Texas.

^††^ p-value for test of linear trendline fitted to VE estimates across ordinal categories of time since vaccination (<2 months = 0; 2–3 months = 1, 4 months = 2, ≥5 months = 3).

During the Omicron-predominant period, VE against COVID-19–associated ED/UC encounters was lower overall compared with that during the Delta-predominant period and waned after the second dose, from 69% within 2 months of vaccination to 37% at ≥5 months after vaccination (p<0.001). Protection increased after a third dose, with VE of 87% among those vaccinated within the past 2 months; however, VE after 3 doses declined to 66% among those vaccinated 4–5 months earlier and 31% among those vaccinated ≥5 months earlier, although the latter estimate is imprecise because few data were available on persons vaccinated for ≥5 months after a third dose. The decreasing trend of VE with increasing time since vaccination was significant (p<0.001).

Among 93,408 eligible hospitalizations, 83,045 (89%) and 10,363 (11%) occurred during the Delta- and Omicron-predominant periods, respectively (Table 3). Among persons hospitalized with COVID-19–like illness, 43% were unvaccinated, 45% had received 2 vaccine doses, and 12% had received 3 doses. The median interval since receipt of the most recent dose before hospitalization was 216 days (IQR = 175–257 days) among those who had received 2 doses and 46 days (IQR = 29–67 days) among those who had received 3 doses, (CDC, unpublished data, 2022).

**TABLE 3:** Characteristics of hospitalizations among adults with COVID-19–like illness,* by mRNA COVID-19 vaccination status^†^ and SARS-CoV-2 test result — 10 states,^§^ August 2021-January 2022^¶^

Characteristic	Total no. (column %)	mRNA COVID-19 vaccination status, no. (row %)			SMD^††^	SARS-CoV-2 test result, no. (row %)		SMD^††^
		Unvaccinated	Vaccinated (2 doses)	Vaccinated (3 doses)**		Negative	Positive	
All hospitalizations	93,408 (100)	40,125 (43)	42,326 (45)	10,957 (12)	—	72,308 (77)	21,100 (23)	—
**Variant predominance period**								
B.1.617.2 (Delta)	83,045 (89)	36,214 (44)	38,707 (47)	8,124 (10)	0.24	65,090 (78)	17,955 (22)	0.15
B.1.1.529 (Omicron)	10,363 (11)	3,911 (38)	3,619 (35)	2,833 (27)		7,218 (70)	3,145 (30)	
**Site**								
Baylor Scott & White Health	17,110 (18)	8,688 (51)	7,182 (42)	1,240 (7)	0.67	13,772 (80)	3,338 (20)	0.43
Columbia University	3,491 (4)	1,494 (43)	1,723 (49)	274 (8)		2,908 (83)	583 (17)	
HealthPartners	1,096 (1)	253 (23)	777 (71)	66 (6)		966 (88)	130 (12)	
Intermountain Healthcare	8,070 (9)	3,741 (46)	3,299 (41)	1,030 (13)		5,643 (70)	2,427 (30)	
Kaiser Permanente Northern California	23,236 (25)	4,967 (21)	13,264 (57)	5,005 (22)		19,952 (86)	3,284 (14)	
Kaiser Permanente Northwest	4,170 (5)	1,702 (41)	1,988 (48)	480 (12)		3,371 (81)	799 (19)	
Regenstrief Institute	25,131 (27)	13,891 (55)	9,415 (37)	1,825 (7)		16,897 (67)	8,234 (33)	
University of Colorado	11,104 (12)	5,389 (49)	4,678 (42)	1,037 (9)		8,799 (79)	2,305 (21)	
**Age group, yrs**								
18–44	17,919 (19)	11,649 (65)	5,550 (31)	720 (4)	0.75	12,998 (73)	4,921 (27)	0.32
45–64	25,620 (27)	13,426 (52)	10,470 (41)	1,724 (7)		18,278 (71)	7,342 (29)	
65–74	20,947 (22)	7,369 (35)	10,471 (50)	3,107 (15)		16,775 (80)	4,172 (20)	
75–84	18,316 (20)	5,003 (27)	9,874 (54)	3,439 (19)		15,215 (83)	3,101 (17)	
≥85	10,606 (11)	2,678 (25)	5,961 (56)	1,967 (19)		9,042 (85)	1,564 (15)	
**Sex**								
Male^§§^	42,175 (45)	18,619 (44)	18,465 (44)	5,091 (12)	0.03	31,609 (75)	10,566 (25)	0.13
Female	51,233 (55)	21,506 (42)	23,861 (47)	5,866 (11)		40,699 (79)	10,534 (21)	
**Race/Ethnicity**								
White, non-Hispanic	60,285 (65)	24,582 (41)	27,842 (46)	7,861 (13)	0.28	47,171 (78)	13,114 (22)	0.16
Hispanic	11,752 (13)	5,559 (47)	5,194 (44)	999 (9)		8,680 (74)	3,072 (26)	
Black, non-Hispanic	10,360 (11)	5,447 (53)	4,200 (41)	713 (7)		8,077 (78)	2,283 (22)	
Other, non-Hispanic^¶¶^	7,199 (8)	2,379 (33)	3,722 (52)	1,098 (15)		5,845 (81)	1,354 (19)	
Unknown	3,812 (4)	2,158 (57)	1,368 (36)	286 (8)		2,535 (67)	1,277 (33)	
Chronic respiratory condition***								
Yes^§§^	59,525 (64)	24,741 (42)	27,360 (46)	7,424 (12)	0.10	46,548 (78)	12,977 (22)	0.06
No	33,883 (36)	15,384 (45)	14,966 (44)	3,533 (10)		25,760 (76)	8,123 (24)	
**Chronic nonrespiratory condition^†††^**								
Yes^§§^	79,433 (85)	31,480 (40)	37,798 (48)	10,155 (13)	0.36	63,475 (80)	15,958 (20)	0.32
No	13,975 (15)	8,645 (62)	4,528 (32)	802 (6)		8,833 (63)	5,142 (37)	
**Immunocompromised status^§§§^**								
Yes^§§^	19,401 (21)	5,988 (31)	9,755 (50)	3,658 (19)	0.33	16,969 (87)	2,432 (13)	0.32
No	74,007 (79)	34,137 (46)	32,571 (44)	7,299 (10)		55,339 (75)	18,668 (25)	
**Total vaccinated**	53,283 (57)	—	42,326 (79)	10,957 (21)		48,518 (91)	4,765 (9)	
**Vaccine product**								
Pfizer-BioNTech	31,460 (59)	—	24,382 (78)	7,078 (22)	—	28,339 (90)	3,121 (10)	0.15
Moderna	21,349 (40)	—	17,850 (84)	3,499 (16)		19,731 (92)	1,618 (8)	
Combination of mRNA products	474 (1)	—	94 (20)	380 (80)		448 (95)	26 (5)	
**No. of doses received (interval from receipt of most recent dose to hospitalization)**								
2 (<2 mos)	1,662 (3)	—	1,662 (100)	—	—	1,591 (96)	71 (4)	0.42
2 (2–3 mos)	3,084 (6)	—	3,084 (100)	—		2,861 (93)	223 (7)	
2 (4 mos)	3,279 (6)	—	3,279 (100)	—		3,045 (93)	234 (7)	
2 (≥5 mos)	34,301 (64)	—	34,301 (100)	—		30,535 (89)	3,766 (11)	
3 (<2 mos)	7,332 (14)	—	—	7,332 (100)		7,111 (97)	221 (3)	
3 (2–3 mos)	3,413 (6)	—	—	3,413 (100)		3,202 (94)	211 (6)	
3 (≥4 mos)	212 (0)	—	—	212 (100)		173 (82)	39 (18)	

**Abbreviations:** ICD-9 = *International Classification of Diseases, Ninth Revision*; ICD-10 *= International Classification of Diseases, Tenth Revision*; SMD = standardized mean or proportion difference.

* Medical events with a discharge code consistent with COVID-19–like illness were included. COVID-19–like illness diagnoses included acute respiratory illness (e.g., COVID-19, respiratory failure, or pneumonia) or related signs or symptoms (cough, fever, dyspnea, vomiting, or diarrhea) using ICD-9 and ICD-10 diagnosis codes. Clinician-ordered molecular assays (e.g., real-time reverse transcription–polymerase chain reaction) for SARS-CoV-2 occurring ≤14 days before to <72 hours after admission were included. Recipients of Janssen vaccine, 1 or >3 doses of an mRNA vaccine, and those for whom 1–13 days had elapsed since receipt of any dose were excluded.

^†^ Vaccination was defined as having received the listed number of doses of an mRNA-based COVID-19 vaccine ≥14 days before the medical event index date, which was the date of respiratory specimen collection associated with the most recent positive or negative SARS-CoV-2 test result before medical event or the admission date if testing only occurred after the admission.

^§^ California, Colorado, Indiana, Minnesota, New York, Oregon, Texas, Utah, Washington, and Wisconsin.

^¶^ Partners contributing data on medical events and estimated date of Omicron predominance were in California (December 21), Colorado (December 19), Indiana (December 26), Minnesota and Wisconsin (December 25), New York (December 18), Oregon (December 24), Texas (December 16), Utah (December 24), and Washington (December 24). The study period began in September 2021 for partners located in Texas.

** Persons categorized as having received 3 vaccine doses include those who have received a third dose in their primary series or have received a booster dose following their 2-dose primary series; the third dose could have been either a 100-*μ*g or 50-*μ*g dose of Moderna vaccine or a 30-*μ*g dose of the Pfizer-BioNTech vaccine.

^††^ An absolute SMD ≥0.20 indicates a nonnegligible difference in variable distributions between medical events for vaccinated versus unvaccinated patients. When calculating SMDs for differences of characteristics across mRNA COVID-19 vaccination status, the SMD was calculated as the average of the absolute value of the SMD for unvaccinated versus vaccinated with 2 doses and the absolute value of the SMD for unvaccinated versus vaccinated with 3 doses. All SMDs are reported as the absolute SMD.

^§§^ Indicates the referent group used for SMD calculations for dichotomous variables.

^¶¶^ Other race includes Asian, Native Hawaiian or other Pacific islander, American Indian or Alaska Native, Other not listed, and multiple races.

*** Chronic respiratory condition was defined using ICD-9 and ICD-10 as the presence of discharge codes for asthma, chronic obstructive pulmonary disease, or other lung disease.

^†††^ Chronic nonrespiratory condition was defined using ICD-9 and ICD-10 as the presence of discharge codes for heart failure, ischemic heart disease, hypertension, other heart disease, stroke, other cerebrovascular disease, diabetes type I or II, other diabetes, metabolic disease, clinical obesity, clinically underweight, renal disease, liver disease, blood disorder, immunosuppression, organ transplant, cancer, dementia, neurologic disorder, musculoskeletal disorder, or Down syndrome.

^§§§^ Immunocompromised status was defined using ICD-9 and ICD-10 as the presence of discharge codes for solid malignancy, hematologic malignancy, rheumatologic or inflammatory disorder, other intrinsic immune condition or immunodeficiency, or organ or stem cell transplant.

During the Delta-predominant period, 2-dose VE against laboratory-confirmed COVID-19–associated hospitalizations declined with increasing time since vaccination and increased after a third dose (Table 2). Among recipients of 3 doses during the Delta-predominant period, VE against COVID-19–associated hospitalizations declined from 96% within 2 months of vaccination to 76% among those vaccinated ≥4 months earlier although the latter estimate is imprecise because few data were available on persons vaccinated for ≥4 months after a third dose during the Delta-predominant period (p<0.001 for test of trend in waning VE).

During the period of Omicron predominance, VE against COVID-19–associated hospitalizations was lower overall and waned with time since vaccination: VE after a second dose declined from 71% within 2 months of vaccination to 54% among those vaccinated ≥5 months earlier (p = 0.01). Among recipients of 3 doses, VE against COVID-19–associated hospitalizations declined from 91% among those vaccinated within the past 2 months to 78% among those vaccinated ≥4 months earlier (p<0.001).

## Discussion

In a multistate analysis of 241,204 ED/UC encounters and 93,408 hospitalizations among adults with COVID-19–like illness during August 26, 2021–January 22, 2022, estimates of VE against laboratory-confirmed COVID-19 were lower during the Omicron-predominant than during the Delta-predominant period, after accounting for both number of vaccine doses received and time since vaccination. During both periods, VE after receipt of a third dose was always higher than VE following a second dose; however, VE waned with increasing time since vaccination. During the Omicron-predominant period, mRNA vaccination was highly effective against both COVID-19–associated ED/UC encounters (VE = 87%) and COVID-19 hospitalizations (VE = 91%) within 2 months after a third dose, but effectiveness waned, declining to 66% for prevention of COVID-19–associated ED/UC encounters by the fourth month after receipt of a third dose and to 78% for hospitalizations by the fourth month after receipt of a third dose. The finding of lower VE for 2 or 3 doses during the Omicron-predominant period is consistent with previous reports from the VISION network and others^¶¶¶^^,^**** (*2*,*7*). Waning of VE after receipt of a third dose of mRNA vaccine has also been observed in Israel (*8*) and in preliminary reports from the VISION Network (*2*). This analysis enhances an earlier VISION Network report (*2*) by extending the Omicron study period to January 22, 2022, providing a more detailed breakdown of time since vaccination, and using an analytic technique that better controls for potential confounding by calendar week and geographic area. By comparing COVID-19 test-positive case-patients with COVID-19 test-negative control patients in the same geographic area and for whom encounter index dates occurred within the same week, bias in VE estimates resulting from temporal and spatial variations in virus circulation and vaccine coverage was reduced.

The findings in this report are subject to at least seven limitations. First, because this study was designed to estimate VE against COVID-19–associated ED/UC visits or hospitalizations, VE estimates from this study do not include COVID-19 infections that were not medically attended. Second, the median interval from receipt of a third dose to medical encounters was 49 days; thus, the observed performance of a third dose is limited to a relatively short period after vaccination. Third, the small number of COVID-19 test-positive patients in the most remote time-since-vaccination groups reduced the precision of the VE estimates for those groups (e.g., ≥5 months). Fourth, variations in waning of VE by age group, immunocompromised status, other indicators of underlying health status, or vaccine product have not yet been examined. This study could not distinguish whether a third dose was received as an additional dose as part of a primary series (as recommended for immunocompromised persons) or as a booster dose after completion of a primary series. Further research should evaluate waning VE of a third primary dose among immunocompromised adults compared with waning of VE after a booster dose among immunocompetent adults. Fifth, despite adjustments to account for differences between unvaccinated and vaccinated persons, VE estimates might have been biased by residual differences between these groups with respect to immunocompromised status and other health conditions, as well as from unmeasured behaviors (e.g., mask use and close contact with persons with COVID-19). For example, insufficient adjustment for immunocompromised status might have biased the estimates of VE downward among persons most remote from receipt of a third dose. Sixth, genetic characterization of patients’ viruses was not available, and analyses relied on dates when the Omicron variant became locally predominant based on surveillance data; therefore, the Omicron period of predominance in this study likely includes some medical encounters associated with the Delta variant. Finally, although the facilities in this study serve heterogeneous populations in 10 states, the findings might not be generalizable to the U.S. population.

These findings underscore the importance of receiving a third dose of mRNA COVID-19 vaccine to prevent both COVID-19–associated ED/UC encounters and COVID-19 hospitalizations among adults. The finding that protection conferred by mRNA vaccines waned in the months after receipt of a third vaccine dose reinforces the importance of further consideration of additional doses to sustain or improve protection against COVID-19–associated ED/UC encounters and COVID-19 hospitalizations. All eligible persons should remain up to date with recommended COVID-19 vaccinations to best protect against COVID-19–associated hospitalizations and ED/UC visits.

**Summary**

**What is already known about this topic?**

Protection against COVID-19 after 2 doses of mRNA vaccine wanes, but little is known about durability of protection after 3 doses.

**What is added by this report?**

Vaccine effectiveness (VE) against COVID-19–associated emergency department/urgent care (ED/UC) visits and hospitalizations was higher after the third dose than after the second dose but waned with time since vaccination. During the Omicron-predominant period, VE against COVID-19–associated ED/UC visits and hospitalizations was 87% and 91%, respectively, during the 2 months after a third dose and decreased to 66% and 78% by the fourth month after a third dose. Protection against hospitalizations exceeded that against ED/UC visits.

**What are the implications for public health practice?**

All eligible persons should remain up to date with recommended COVID-19 vaccinations to best protect against COVID-19–associated hospitalizations and ED/UC visits.

## Footnotes

* On November 29, 2021, CDC initially recommended a third dose of mRNA vaccine for all adults 6 months after receipt of the second primary series mRNA COVID-19 vaccine dose. The third dose of the BNT162b2 (Pfizer-BioNTech) vaccine was the same dosage as the primary series; however, the third dose of the mRNA-1273 (Moderna) vaccine was a reduced dosage compared with the primary series for all but immunocompromised persons; the third dose was either a 100-*μ*g or 50-*μ*g dose of Moderna vaccine or a 30-*μ*g dose of the Pfizer-BioNTech vaccine. On January 4, 2022, CDC amended the interval to 5 months after receipt of the second dose for recipients of the Pfizer-BioNTech vaccine. On January 7, 2022, CDC amended the interval to 5 months for recipients of the Moderna vaccine. CDC recommends the Pfizer-BioNTech booster at 5 months, and an additional primary dose for certain immunocompromised children (https://www.cdc.gov/media/releases/2022/s0104-Pfizer-Booster.html). CDC recommends the Moderna booster at 5 months after completion of the primary series. (https://www.cdc.gov/media/releases/2022/s0107-moderna-booster.html). CDC recommends additional primary doses for some immunocompromised persons (https://www.cdc.gov/coronavirus/2019-ncov/vaccines/recommendations/immuno.html).

†
https://papers.ssrn.com/sol3/papers.cfm?abstract_id=3961378

§ Funded by CDC, the VISION Network includes Baylor Scott & White Health (Texas), Columbia University Irving Medical Center (New York), HealthPartners (Minnesota and Wisconsin), Intermountain Healthcare (Utah), Kaiser Permanente Northern California (California), Kaiser Permanente Northwest (Oregon and Washington), Regenstrief Institute (Indiana), and University of Colorado (Colorado).

¶ Partners contributing data on medical events (and estimated dates of Omicron predominance) were as follows: California (December 21), Colorado (December 19), Indiana (December 26), Minnesota and Wisconsin (December 25), New York (December 18), Oregon (December 24), Texas (December 16), Utah (December 24), and Washington (December 24). The study period began in September 2021 for partners located in Texas.

** ED data at Columbia University Irving Medical Center and HealthPartners exclude encounters that were transferred to an inpatient setting.

†† COVID-19–like illness diagnoses included acute respiratory illness (e.g., COVID-19, respiratory failure, or pneumonia) or related signs or symptoms (cough, fever, dyspnea, vomiting, or diarrhea) using diagnosis codes from the *International Classification of Diseases, Ninth Revision* and *International Classification of Diseases, Tenth Revision*.

§§
https://www.fda.gov/news-events/press-announcements/coronavirus-covid-19-update-fda-authorizes-additional-vaccine-dose-certain-immunocompromised

¶¶ VE was calculated as [1 − odds ratio] x 100%, estimated using a test-negative design, which can be considered a case-control design in which case-patients were those whose outcome was confirmed COVID-19 and control patients were those with COVID-19–like illness and negative SARS-CoV-2 test results. All VE models were conditioned on calendar week and geographic area and adjusted for age, local virus circulation (percentage of SARS-CoV-2–positive results from testing within the counties surrounding the facility on the date of the encounter), propensity to be vaccinated (calculated separately for each VE estimate), and other patient and facility characteristics. Generalized boostered regression tree methods were used to estimate the propensity to be vaccinated based on sociodemographic characteristics, underlying medical conditions, and facility characteristics.

*** Immunocompromising conditions were derived from lists used in previous studies of large hospital-based or administrative databases and included the following conditions: 1) solid malignancies, 2) hematologic malignancies, 3) rheumatologic or inflammatory disorders, 4) other intrinsic immune conditions or immunodeficiencies, and 5) organ or stem cell transplants. https://www.cdc.gov/mmwr/volumes/70/wr/mm7044e3.htm?s_cid=mm7044e3_w

††† The index date for each medical visit was defined as either the date of collection of a respiratory specimen associated with the most recent positive or negative SARS-CoV-2 test result before the medical visit or the date of the medical visit (if testing occurred only after the admission or visit date).

§§§ 45 C.F.R. part 46; 21 C.F.R. part 56.

¶¶¶
https://www.medrxiv.org/content/10.1101/2021.12.14.21267615v1

****
https://www.medrxiv.org/content/10.1101/2021.12.20.21267966v3
